# Multi-Omic Approaches to Classify, Predict, and Treat Acute Leukemias

**DOI:** 10.3390/cancers15041049

**Published:** 2023-02-07

**Authors:** Maria Hernandez-Valladares

**Affiliations:** 1 Department of Physical Chemistry, University of Granada, 18071 Granada, Spain; mariahv@ugr.es; Tel.: +34-958241000 (ext. 20284); 2 Instituto de Investigación Biosanitaria ibs.GRANADA, 18012 Granada, Spain; 3 Institute of Biotechnology, School of Sciences, University of Granada, 18071 Granada, Spain

## 1. Current ALL and AML Classification and Therapeutical Strategies

Acute lymphoblastic leukemia (ALL) is the most common childhood cancer, in which nearly 5% of the cases are diagnosed before the first year of age [1]. Around 75% of the pediatric ALL cases are characterized by a high frequency of lysine methyltransferase 2A (*KTM2A*, previously known as *MLL*) gene rearrangement *KMT2A*-r, which is associated with neprilysin (CD10)-negative immature B-cell precursor phenotype and a very poor prognosis [2]. Recently, an unbiased molecular classification of *KTM2A*-r based on transcription factors (iroquois-class homeodomain protein IRX and homeobox protein HOXA), fusion partners, and corresponding stages of B-lymphopoietic and hemato-endothelial development has been suggested for genomic-driven diagnostics and potential therapeutic strategies in infant ALL [3]. Interestingly, the combined mutations of KMT2D and phosphatidylinositol 3,4,5-triphosphate 3-phosphatase and dual-specificity protein phosphatase (PTEN) defined a group of T-cell lymphoblastic lymphoma patients with a high incidence of relapse [4]. Nearly 70% of childhood B-cell ALL (B-ALL) is currently characterized by well-known cytogenetic abnormalities, which are associated with good or poor outcomes [5]. Recently, genomic approaches have identified new genetic features among the remaining 30%, including double homeobox protein 4 (*DUX4)*-rearranged (*DUX4*-r), tyrosine-protein kinase ABL (ABL)-class fusions, and myocyte-specific enhancer factor 2 D (*MEF2D*)-rearranged (*MED2D*-r) [6,7,8]. A short while ago, whole-genome sequencing (WGS) on 210 childhood B-ALL cases detected 294 subtype-defining genetic abnormalities in 96% of patients. These included fusions affecting genes in the mitogen-activated protein (MAP) kinase pathway and improved detection of *DUX4*-r [9]. Last year, RNA-sequencing (RNA-seq) technology contributed remarkably to ALL classifications. RNA-seq data from leukemic cells of 1,988 patients with B-ALL provided a revised taxonomy of B-ALL, incorporating 23 subtypes defined by chromosomal rearrangements, sequence mutations, or heterogeneous genomic alterations [10]. To classify RNA-seq data according to 18 of these 23 subtypes, ALLSorts software has recently been developed to analyze B-ALL gene expression data and attribute study samples to the described subtypes [11].

ALL treatment typically involves several phases: induction (e.g., vincristine, prednisone, and anthracycline), prophylaxis (e.g., methotrexate, cytarabine, and radiation therapy), consolidation (allogeneic stem cell transplant (SCT)) and maintenance (e.g., methotrexate and 6-mercaptopurine). Targeted therapy protocols including the use of tyrosine kinase inhibitors to Philadelphia chromosome-positive ALL patients and of immunotherapeutic agents such as antibody and chimeric antigen receptor (CAR) T-cell therapies are now being examined to be part of the upfront setting [12,13].

Acute myeloid leukemia (AML) is an aggressive and heterogeneous hematological cancer. Although most patients with newly diagnosed AML achieve complete remission (CR) after intensive induction and consolidation therapy, more than half of them relapse within the next three years [14,15]. Because of the advances in cytogenetics, molecular biology, next-generation sequencing (NGS), and an increasing number of prognostic markers and therapeutic targets (i.e., Fms-like tyrosine kinase 3 (FLT3), B-cell lymphoma 2 (BCL2), and isocitrate dehydrogenase 1 and 2 (IDH1/2)), AML cases are now classified according to the World Health Organization (WHO) system that integrates clinical, molecular/genetic, and pathologic parameters, providing the separation of AML with defining genetic abnormalities from AML defined by differentiation [16]. This WHO classification has been recently supported by transcriptomics and differentiation hierarchies in the largest cohort of AML patients in China [17]. The relevance of genomic characterization has also been reflected in the new International Consensus Classification (ICC, [18]) and the European LeukemiaNet (ELN) risk stratification [19]. Recently, the molecular genetic characterization of Philadelphia chromosome-positive AML has been published, which showed that patients with this type of AML shared similar genetic profiles and clinical outcomes with those with chronic myeloid leukemia in myeloid blast crisis (CML-MBC) [20]. Within a national context, the cooperative initiative PETHEMA has established the first nationwide diagnostic network to provide standardized NGS studies for AML patients [21]. Moreover, it reported a distinct molecular profile between age groups at diagnosis and sex. In such a national effort, the clinical validation of genomic classifications in the PETHEMA cohort consisting of seven reference laboratories is carried out to demonstrate or adjust the correlation of the molecular subgroups with clinical prognosis.

Besides intensive induction and consolidation therapy, AML treatment has improved in recent years, and several new therapeutic options have been approved. Most of them include mutation-specific approaches (e.g., gilteritinib for AML patients with activating *FLT3* mutations), combined epigenetic therapy with the BCL-2 inhibitor venetoclax, immunotherapy or restricted approaches for AML with myeloid-related changes (AML-MRC), or therapy-related AML (CPX-351) cases [22].

## 2. Single-Omics and Integration of Multi-Omics

The genetic and clinical heterogeneity of ALL and AML cases represent a challenge even for the new therapies. Moreover, relapse and treatment resistance seriously hinder ALL and AML treatment. To understand the biology underlying those differences, omic sciences have increasingly been utilized to produce useful knowledge.

Multi-omic analyses of 49 childhood ALL cell lines using proteomics, transcriptomics, and pharmacoproteomic characterization (arranged as a database for the interactive online Functional Omics Resource of ALL (FORALL), at https://proteomics.se/forall accessed on 8 January 2023) identified the diacylglycerol-analog bryostatin-1 as a therapeutic candidate in the myocyte enhancer factor 2D *(MEF2D*)*-*heterogeneous nuclear ribonucleoprotein U-like protein 1 (*HNRNPUL1*) fusion high-risk subtype, for which this drug activates pro-apoptotic mitogen-activated protein kinase (ERK) signaling associated with the molecular mediators of pre-B-cell negative selection [23].

To uncover the molecular changes allowing AML cells to escape treatment, two proteomic studies with liquid chromatography–mass spectrometry (LC–MS) and serial time-point samples during the disease progression of patients have shown that the proteomic profile at relapse is enriched for mitochondrial ribosomal proteins and subunits of the respiratory chain complex, indicative of reprogrammed energy metabolism from diagnosis to relapse [14,24]. Using a proteogenomic strategy, the most recent study on the latter detected 370 novel peptides, which represent a promising repertoire in the search for biomarkers and tumor-specific druggable targets. A multi-omic approach involving genomics, transcriptomics, LC–MS proteomics, and phosphoproteomics identified examples of post-transcriptionally regulated proteins and phosphorylation events both in all studied AML samples and also in patients with recurrent AML driver mutations (e.g., samples with *IDH1/2* mutations displayed a high expression of the 2-oxoglutarate–dependent histone demethylases KDM4A/B/C, despite no changes in messenger RNA levels for these genes, and samples with FLT3-tyrosine kinase domain (TKD) mutationsassociated with the activation of the SRC-family tyrosine kinases FGR and HCK) [25]. Using systems medicine and multi-omics, an approach involving transcriptomics, proteomics, and metabolomics identified new molecular references such as mir-484, miR-519d-3p, activin receptor type-1 (ACVR1), receptor-type tyrosine-protein phosphatase gamma (PTPRG), PR domain zinc finger protein 14 (PRDM14), trans-acting T-cell-specific transcription factor GATA-3 (GATA3), and amino acid derivatives [26].

Altogether, these studies show us the importance and strength of single- and multi-omic approaches to decode the complex and heterogenous molecular basis of acute leukemias and to improve the current treatment and survival of ALL and AML patients.

## 3. Implementation and Future Use

In the past few decades, single-omic strategies have been used for research leading to ALL- and AML-related biomarker discovery. Genomic approaches have described several causative variants that are now part of international diagnosis guidelines. However, the integration of genome sequencing into a healthcare setting still lags behind. Nevertheless, examples of genomic incorporation, such as the reported initiative across multiple clinical entities of rare diseases [27] and the PETHEMA initiative described earlier, should promote the implementation of genomic approaches into clinical diagnosis. To facilitate the identification of genomic alterations of clinical significance in ALL and encourage the implementation of RNA-seq analysis in the clinic, the RaScALL platform (codes and scripts) is publicly available from the GitHub repository (https://github.com/j-rehn/RaScALL accessed on 10 January 2023) [28].

**Figure 1:**
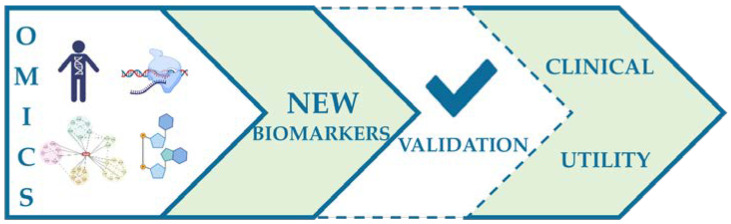
Single- and multi-omic workflow (e.g., genomics, transcriptomics, proteomics, and metabolomics) from new biomarker discovery to new biomarker applications in the clinic.

Numerous single-omic studies with patient cohorts have revealed a high number of leukemia biomarkers [29,30,31]. Regarding MS-based omic studies for biomarker discovery, very few of them have undergone validation to support their use in the clinic (Figure 1). The real bottleneck when considering the clinical use of new biomarkers is their validation using external cohorts. Because of the remarkable efforts and funds employed in both single- and multi-omic projects, it is hoped that stronger validation initiatives and closer collaborations at national and international levels can be established soon so that the introduction and use of new biomarkers in the clinic unveil more accurate diagnostic and treatment tools to defeat acute leukemias.

Multi-omic approaches that integrate the data from distinct levels of the cellular organization, i.e., from genes to metabolites, have recently been reported, which offer new perspectives for innovation in ALL and AML diagnostics and therapeutics. Multi-omic strategies bring together researchers with different expertise, including data managers and bioinformaticians, to provide protein and metabolite targets of specific genomic backgrounds. The incorporation of more clinicians in research teams appears to be more necessary than ever to transfer clinical innovation into practice.

## References

[1] Downing J.R., Wilson R.K., Zhang J., Mardis E.R., Pui C.H., Ding L., Ley T.J., Evans W.E. (2012). The Pediatric Cancer Genome Project. Nat. Genet..

[2] Tomizawa D., Miyamura T., Imamura T., Watanabe T., Moriya Saito A., Ogawa A., Takahashi Y., Hirayama M., Taki T., Deguchi T. (2020). A risk-stratified therapy for infants with acute lymphoblastic leukemia: A report from the JPLSG MLL-10 trial. Blood.

[3] Isobe T., Takagi M., Sato-Otsubo A., Nishimura A., Nagae G., Yamagishi C., Tamura M., Tanaka Y., Asada S., Takeda R. (2022). Multi-omics analysis defines highly refractory RAS burdened immature subgroup of infant acute lymphoblastic leukemia. Nat. Commun..

[4] Khanam T., Sandmann S., Seggewiss J., Ruether C., Zimmermann M., Norvil A.B., Bartenhagen C., Randau G., Mueller S., Herbrueggen H. (2021). Integrative genomic analysis of pediatric T-cell lymphoblastic lymphoma reveals candidates of clinical significance. Blood.

[5] Schwab C.J., Murdy D., Butler E., Enshaei A., Winterman E., Cranston R.E., Ryan S., Barretta E., Hawking Z., Murray J. (2022). Genetic characterisation of childhood B-other-acute lymphoblastic leukaemia in UK patients by fluorescence in situ hybridisation and Multiplex Ligation-dependent Probe Amplification. Br. J. Haematol..

[6] Gu Z., Churchman M., Roberts K., Li Y., Liu Y., Harvey R.C., McCastlain K., Reshmi S.C., Payne-Turner D., Iacobucci I. (2016). Genomic analyses identify recurrent MEF2D fusions in acute lymphoblastic leukaemia. Nat. Commun..

[7] Schwab C., Cranston R.E., Ryan S.L., Butler E., Winterman E., Hawking Z., Bashton M., Enshaei A., Russell L.J., Kingsbury Z. (2022). Integrative genomic analysis of childhood acute lymphoblastic leukaemia lacking a genetic biomarker in the UKALL2003 clinical trial. Leukemia.

[8] Zhang J., McCastlain K., Yoshihara H., Xu B., Chang Y., Churchman M.L., Wu G., Li Y., Wei L., Iacobucci I. (2016). Deregulation of DUX4 and ERG in acute lymphoblastic leukemia. Nat. Genet..

[9] Ryan S.L., Peden J.F., Kingsbury Z., Schwab C.J., James T., Polonen P., Mijuskovic M., Becq J., Yim R., Cranston R.E. (2023). Whole genome sequencing provides comprehensive genetic testing in childhood B-cell acute lymphoblastic leukaemia. Leukemia.

[10] Gu Z., Churchman M.L., Roberts K.G., Moore I., Zhou X., Nakitandwe J., Hagiwara K., Pelletier S., Gingras S., Berns H. (2019). PAX5-driven subtypes of B-progenitor acute lymphoblastic leukemia. Nat. Genet..

[11] Schmidt B., Brown L.M., Ryland G.L., Lonsdale A., Kosasih H.J., Ludlow L.E., Majewski I.J., Blombery P., Ekert P.G., Davidson N.M. (2022). ALLSorts: An RNA-Seq subtype classifier for B-cell acute lymphoblastic leukemia. Blood Adv..

[12] Biondi A., Gandemer V., De Lorenzo P., Cario G., Campbell M., Castor A., Pieters R., Baruchel A., Vora A., Leoni V. (2018). Imatinib treatment of paediatric Philadelphia chromosome-positive acute lymphoblastic leukaemia (EsPhALL2010): A prospective, intergroup, open-label, single-arm clinical trial. Lancet Haematol..

[13] Lee D.W., Kochenderfer J.N., Stetler-Stevenson M., Cui Y.K., Delbrook C., Feldman S.A., Fry T.J., Orentas R., Sabatino M., Shah N.N. (2015). T cells expressing CD19 chimeric antigen receptors for acute lymphoblastic leukaemia in children and young adults: A phase 1 dose-escalation trial. Lancet.

[14] Aasebo E., Berven F.S., Hovland R., Doskeland S.O., Bruserud O., Selheim F., Hernandez-Valladares M. (2020). The Progression of Acute Myeloid Leukemia from First Diagnosis to Chemoresistant Relapse: A Comparison of Proteomic and Phosphoproteomic Profiles. Cancers.

[15] Aasebo E., Berven F.S., Bartaula-Brevik S., Stokowy T., Hovland R., Vaudel M., Doskeland S.O., McCormack E., Batth T.S., Olsen J.V. (2020). Proteome and Phosphoproteome Changes Associated with Prognosis in Acute Myeloid Leukemia. Cancers.

[16] Khoury J.D., Solary E., Abla O., Akkari Y., Alaggio R., Apperley J.F., Bejar R., Berti E., Busque L., Chan J.K.C. (2022). The 5th edition of the World Health Organization Classification of Haematolymphoid Tumours: Myeloid and Histiocytic/Dendritic Neoplasms. Leukemia.

[17] Cheng W.Y., Li J.F., Zhu Y.M., Lin X.J., Wen L.J., Zhang F., Zhang Y.L., Zhao M., Fang H., Wang S.Y. (2022). Transcriptome-based molecular subtypes and differentiation hierarchies improve the classification framework of acute myeloid leukemia. Proc. Natl. Acad. Sci. USA.

[18] Arber D.A., Orazi A., Hasserjian R.P., Borowitz M.J., Calvo K.R., Kvasnicka H.M., Wang S.A., Bagg A., Barbui T., Branford S. (2022). International Consensus Classification of Myeloid Neoplasms and Acute Leukemias: Integrating morphologic, clinical, and genomic data. Blood.

[19] Dohner H., Wei A.H., Appelbaum F.R., Craddock C., DiNardo C.D., Dombret H., Ebert B.L., Fenaux P., Godley L.A., Hasserjian R.P. (2022). Diagnosis and management of AML in adults: 2022 recommendations from an international expert panel on behalf of the ELN. Blood.

[20] Zhou Q., Zhao D., Eladl E., Capo-Chichi J.M., Kim D.D.H., Chang H. (2023). Molecular genetic characterization of Philadelphia chromosome-positive acute myeloid leukemia. Leuk. Res..

[21] Sargas C., Ayala R., Larrayoz M.J., Chillon M.C., Carrillo-Cruz E., Bilbao-Sieyro C., Prados de la Torre E., Martinez-Cuadron D., Rodriguez-Veiga R., Boluda B. (2023). Molecular Landscape and Validation of New Genomic Classification in 2668 Adult AML Patients: Real Life Data from the PETHEMA Registry. Cancers.

[22] Fleischmann M., Schnetzke U., Hochhaus A., Scholl S. (2021). Management of Acute Myeloid Leukemia: Current Treatment Options and Future Perspectives. Cancers.

[23] Leo I.R., Aswad L., Stahl M., Kunold E., Post F., Erkers T., Struyf N., Mermelekas G., Joshi R.N., Gracia-Villacampa E. (2022). Integrative multi-omics and drug response profiling of childhood acute lymphoblastic leukemia cell lines. Nat. Commun..

[24] Stratmann S., Vesterlund M., Umer H.M., Eshtad S., Skaftason A., Herlin M.K., Sundstrom C., Eriksson A., Hoglund M., Palle J. (2022). Proteogenomic analysis of acute myeloid leukemia associates relapsed disease with reprogrammed energy metabolism both in adults and children. Leukemia.

[25] Kramer M.H., Zhang Q., Sprung R., Day R.B., Erdmann-Gilmore P., Li Y., Xu Z., Helton N.M., George D.R., Mi Y. (2022). Proteomic and phosphoproteomic landscapes of acute myeloid leukemia. Blood.

[26] Kelesoglu N., Kori M., Turanli B., Arga K.Y., Yilmaz B.K., Duru O.A. (2022). Acute Myeloid Leukemia: New Multiomics Molecular Signatures and Implications for Systems Medicine Diagnostics and Therapeutics Innovation. OMICS.

[27] Stranneheim H., Lagerstedt-Robinson K., Magnusson M., Kvarnung M., Nilsson D., Lesko N., Engvall M., Anderlid B.M., Arnell H., Johansson C.B. (2021). Integration of whole genome sequencing into a healthcare setting: High diagnostic rates across multiple clinical entities in 3219 rare disease patients. Genome Med..

[28] Rehn J., Mayoh C., Heatley S.L., McClure B.J., Eadie L.N., Schutz C., Yeung D.T., Cowley M.J., Breen J., White D.L. (2022). RaScALL: Rapid (Ra) screening (Sc) of RNA-seq data for prognostically significant genomic alterations in acute lymphoblastic leukaemia (ALL). PLoS Genet..

[29] Aasebo E., Forthun R.B., Berven F., Selheim F., Hernandez-Valladares M. (2016). Global Cell Proteome Profiling, Phospho-signaling and Quantitative Proteomics for Identification of New Biomarkers in Acute Myeloid Leukemia Patients. Curr. Pharm. Biotechnol..

[30] Bardelli V., Arniani S., Pierini V., Di Giacomo D., Pierini T., Gorello P., Mecucci C., La Starza R. (2021). T-Cell Acute Lymphoblastic Leukemia: Biomarkers and Their Clinical Usefulness. Genes.

[31] Prada-Arismendy J., Arroyave J.C., Rothlisberger S. (2017). Molecular biomarkers in acute myeloid leukemia. Blood Rev..

